# Systematic comparison of multi-omics survival models reveals a widespread lack of noise resistance

**DOI:** 10.1016/j.crmeth.2023.100461

**Published:** 2023-04-24

**Authors:** David Wissel, Daniel Rowson, Valentina Boeva

**Affiliations:** 1 ETH Zurich, Department of Computer Science, Zurich, Switzerland; 2 University of Zurich, Department of Molecular Life Sciences, Zurich, Switzerland; 3 SIB Swiss Institute of Bioinformatics, Lausanne, Switzerland; 4 Université de Paris UMR-S1016, Institut Cochin, Inserm U1016, Paris, France

**Keywords:** survival analysis, cancer, statistical models, deep learning models, multi-modal integration, multi-omics, noise resistance

## Abstract

As observed in several previous studies, integrating more molecular modalities in multi-omics cancer survival models may not always improve model accuracy. In this study, we compared eight deep learning and four statistical integration techniques for survival prediction on 17 multi-omics datasets, examining model performance in terms of overall accuracy and noise resistance. We found that one deep learning method, mean late fusion, and two statistical methods, *PriorityLasso* and *BlockForest*, performed best in terms of both noise resistance and overall discriminative and calibration performance. Nevertheless, all methods struggled to adequately handle noise when too many modalities were added. In summary, we confirmed that current multi-omics survival methods are not sufficiently noise resistant. We recommend relying on only modalities for which there is known predictive value for a particular cancer type until models that have stronger noise-resistance properties are developed.

## Highlights

• Multi-omics survival models are compared across The Cancer Genome Atlas (TCGA) datasets
• Only one model performs better than a model trained using clinical data alone
• *PriorityLasso* is recommended when the informativeness of modalities is uncertain

## Motivation

With decreasing costs of high-throughput sequencing, more and more studies have started to provide multi-omics molecular profiles of patients with cancer. This has led to various developments of novel survival analysis approaches integrating these heterogeneous molecular and clinical groups of variables. Although some of these methods have reached state-of-the-art results in cancer survival prediction, many have demonstrated a decay in performance when integrating larger numbers of omics modalities.

## Graphical abstract

**Figure:**
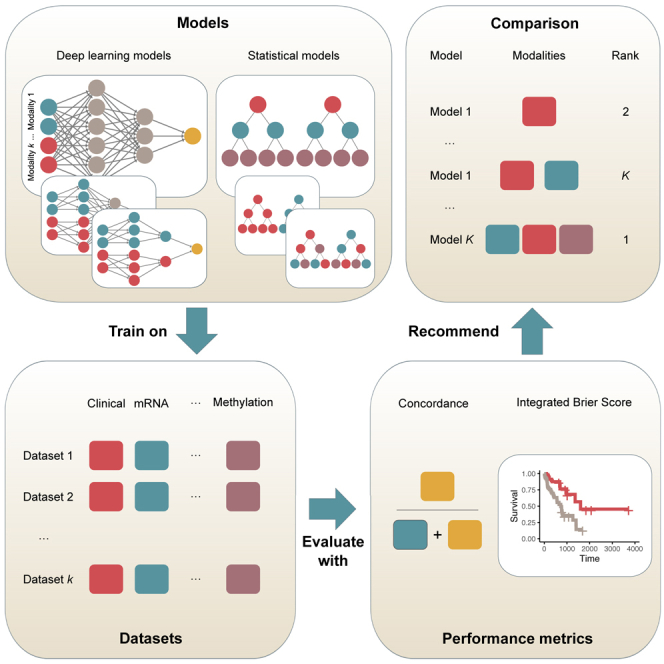

## Teaser

Wissel et al. perform an empirical comparison of 12 models for predicting survival from multi-omics data on 17 datasets from The Cancer Genome Atlas (TCGA). The authors show that all considered methods suffer from a lack of noise resistance. The study emphasizes the need for more noise-resistant multi-omics survival methods.

## Introduction

The accurate prediction of survival times is essential for clinicians and researchers to make treatment choices and identify which factors are driving survival outcomes. The Cox Proportional Hazards (Cox PH) model^1^^,^^2^ is still the *de facto* standard model for survival analysis today, despite proposals for various other methods such as random survival forests,^3^ boosting,^4^ and neural networks.^5^^,^^6^ Survival analysis of cancer patients can be particularly challenging due to the heterogeneous nature of the disease, even for patients suffering from the same cancer type.^7^^,^^8^^,^^9^ With the advent of high-throughput sequencing technologies, researchers have hoped to be able to leverage information inherent in molecular data such as gene expression, DNA methylation, genetic mutations, and others (jointly referred to as multi-omics) to help mitigate this heterogeneity.

Even using the wealth of newly available sequencing data in large-scale projects such as The Cancer Genome Atlas (TCGA),^10^ significant improvements in performance in cancer survival analysis as measured by discriminative performance metrics such as Harrell’s concordance (Harrell’s C)^11^ have been elusive. Herrmann et al.^12^ showed that the Cox PH model using (only) clinical data outperformed all but one statistical model, even when these were specifically designed to integrate multi-omics data.

There have been numerous proposals for statistical models that perform multi-omics integration in the context of cancer survival analysis. Hornung and Wright^13^ proposed five variations of the random survival forest (RSF) algorithm,^3^ which modify the split point selection by considering the group structure of the input data in various ways. *BlockForest*, the best performing of their five proposed variants, significantly outperformed RSF^13^ and was shown capable of slightly outperforming the clinical-only Cox PH model on TCGA by Herrmann et al.^12^ in terms of Harrell’s C. Boulesteix et al.^14^ proposed a modified Lasso-regularized Cox PH model that scales the Lasso penalty with a group-specific penalty factor that can be chosen through *a priori* knowledge or using cross-validation. The authors showed that their new model, termed *IPF-Lasso*, outperformed a Lasso-regularized Cox PH model in simulations and on TCGA in terms of Integrated Brier score (IBS).^15^ Klau et al.^16^ introduced a sequential Lasso-regularized Cox PH model approach based on offsetting, called *PriorityLasso*, which considers input modality groups one at a time and uses the previous model prediction as an unregularized offset for the model fit with the next input group. Their model outperformed a Lasso-regularized Cox PH model on a cancer dataset in terms of IBS and has offered clinicians the chance of influencing which (groups of) variables should be preferentially included in the model by assigning them a higher priority order.^16^

In addition to ample work on statistical models, researchers have also been interested in applying deep learning to multi-omics survival analysis tasks. Deep learning models for multi-omics integration generally rely on one of late, intermediate, or early fusion.^17^^,^^18^ Further, deep learning models commonly differ between those training on multiple cancers simultaneously (pan-cancer) or on each cancer separately.

Pan-cancer models aim to leverage commonalities between cancer types for improved performance. Cheerla and Gevaert^19^ proposed an intermediate fusion architecture to predict cancer survival by integrating gene expression, miRNA, clinical data, and whole-slide images by using a similarity loss combined with mean-pooling to combine the different modalities. Their model benefited (that is, exhibited an increased Harrell’s C) from pan-cancer training relative to training solely on each cancer type for most considered cancers. Zhang et al.^20^ developed a multi-task pan-cancer intermediate fusion multi-omics model that integrates the modality-specific representations learned by one neural network per modality using one top-level variational autoencoder. Their model, deemed *OmiEmbed*, outperformed *DeepSurv* and a Cox PH model based on the dimensionality-reduced input data in terms of Harrell’s C and the IBS on the full TCGA dataset by integrating gene expression, DNA methylation, and miRNA. Vale-Silva and Rohr^21^ presented a pan-cancer multimodal intermediate fusion neural network, *Multi-Surv*, which uses max-pooling to integrate the representations constructed by a separate neural network per modality, in addition to using a non-proportional hazards loss by assuming discrete follow-up times.^22^ In the work of Vale-Silva and Rohr,^21^ *Multi-Surv* outperformed a regularized Cox PH model, RSF, *DeepSurv*^5^ and *DeepHit*^23^ in unimodal settings and was able to achieve state-of-the-art results when integrating multiple modalities.

Per-cancer models instead fit individual models per cancer type to better account for differences between cancer types. Tong et al.^24^ explored multimodal intermediate fusion autoencoders for the integration of multi-omics data in breast cancer survival. They proposed two architectures, each of which fits a dedicated autoencoder per modality. Their second architecture, based on concatenating the modality-specific representations, called *ConcatAE*, performed best on TCGA breast cancer when integrating DNA methylation and miRNA while using principal component analysis (PCA) for dimensionality reduction. Chai et al.^25^ employed an early fusion approach combined with a denoising autoencoder and found that a Cox PH model trained on its latent space outperformed both regularized Cox PH model variants and other dimensionality reduction techniques such as using PCA, as well as intermediate fusion-based autoencoders.

Our work aims to fill a gap in the literature by focusing on the noise resistance of multi-omics survival methods. In particular, we analyzed published approaches and found that, to the best of our knowledge, no previous work had exhaustively compared diverse integration techniques for multi-omics survival analysis with a focus on their resistance to noise on the modality level. Despite selected published work exhibiting performance decreases when a high number of modalities was integrated, there has been little to no further exploration of this phenomenon.^13^^,^^21^^,^^26^ Our work thus focuses on a rigorous and exhaustive comparison of integration techniques for multi-omics survival models, with a particular focus on how each of those integration techniques handles noise.

We performed a large-scale empirical comparison of eight deep learning and four statistical multi-omics integration techniques. Though none of the considered techniques exhibited perfect noise resistance, late fusion and intermediate fusion approaches strongly outperformed early fusion approaches due to their superior ability to leverage low-dimensional but highly predictive modalities. More broadly, researchers should choose models taking the group structure into account, as these outperformed models discarding the group structure throughout our work.

## Results

### Research questions and matching experiments

First, we were interested in which multi-omics integration survival methods performed best in the low-noise setting, in which all modalities to be integrated provided relatively high predictive value. To answer this question, we compared the performance of different methods using only two input modalities: clinical data and gene expression. This combination was shown to be effective in previous research, and initial experiments indicated that these modalities outperformed others in the unimodal setting (Table S2).^13^^,^^21^^,^^26^

We explored which integration methods were most effective in settings where certain modalities provided little or no information about patient survival. To benchmark the considered integration methods in this partially noisy setting, we first ran all considered methods using all seven available modalities in the TCGA database and compared their performance to those using only clinical and gene expression data. This setting has in the past often yielded reduced performance.^13^^,^^21^^,^^26^To further test the performance of the different methods, we created additional input modalities by generating data from a standard Gaussian distribution with a dimensionality of 10,000. We then evaluated the ability of each method to integrate clinical data and gene expression with one, three, and five of these noise modalities to measure how wellB each integration technique could filter out pure noise.

Last, our work investigated which integration methods were best able to leverage highly informative modalities despite the presence of noisy modalities. To answer this research question, we repeated the latter simulated benchmark, except that, in each experiment, we also added the true survival time T, as an additional “clinical” variable, simulating an extremely informative but low-dimensional modality. We again considered the combination of clinical data and gene expression with m simulated modalities (m=1,3,5). In the final experiment, we found that adding observed survival times as a feature often resulted in numerical instability when calculating survival functions using the Breslow estimator.^27^ This was likely due to the extreme log hazard ratios assigned to the noisy observed survival times. As a result, we excluded the IBS from this experiment and only evaluated Antolini’s concordance (Antolini’s C), which was not affected by this issue.

Further details regarding our datasets, experiments, and training details (e.g., hyperparameter tuning) may be found in STAR Methods.

Throughout the text, we refer to some integration methods using shortened names. We refer to the four considered statistical models, namely *BlockForest*, *PriorityLasso*, RSF, and Elastic net using their full names. We refer to deep learning models as *Early*, *Intermediate*, or *Late* based on whether they use early, intermediate or late fusion. In addition, we add the type of integration used by each deep learning method in parentheses. In particular, *Intermediate (Mean)* refers to an intermediate fusion approach that uses mean pooling, while *Intermediate (Concat)* and *Intermediate (Max)* refer to a concatenation operation and max pooling as the methods to fuse all modalities. *Intermediate (Attention)* and *Intermediate (Embrace)* use attention and the Embrace method to fuse the intermediate modality representations (for details see STAR Methods). Further, early fusion discards the modality structure, thus we denote it only as *Early*. Lastly, for late fusion, we distinguish *Late (Mean)* and *Late (MoE)*, which respectively use a mean pooling and a weighted mean (based on a gating network) operation to fuse the partial hazards predictions of each modality network. An exhaustive description of all methods may be found in STAR Methods.

### Group-aware integration methods outperform naive integration methods

**Figure 1:**
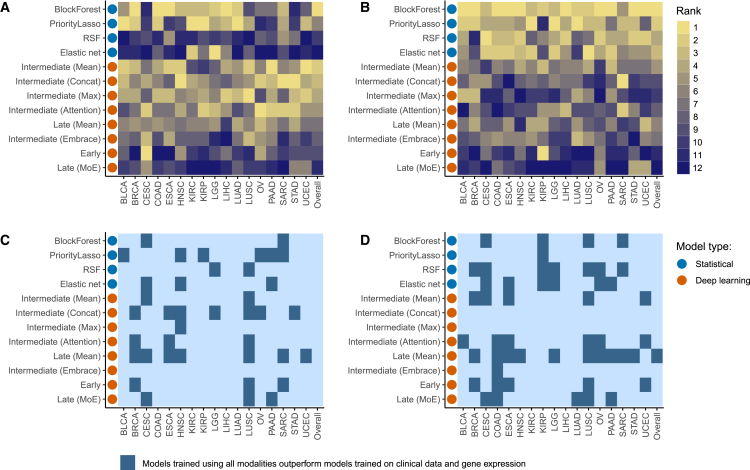
The performance of all considered methods varied widely across datasets (A and B) Statistical methods outperformed deep learning methods in terms of calibration (measured by the Integrated Brier score [IBS]) for multi-omics survival analysis when trained on clinical data and gene expression. Rank of all models on each dataset when integrating clinical data and gene expression in terms of mean Antolini’s C and mean IBS across splits. Lower ranks indicate better performance. (A) Antolini’s concordance (Antolini’s C). (B) IBS. For full cancer-type abbreviations, please refer to Table S3. (C and D) Using fewer but more predictive modalities generally led to improved performance for most models. Model performance in terms of mean Antolini’s C and mean IBS across splits as a function of the number of modalities used for training: models fitted on clinical data and gene expression compared to all modalities. The dark color indicates that the model in question performed better for a particular dataset when trained on all modalities compared to only clinical data and gene expression modalities used as input. (C) Comparison for Antolini’s concordance. (D) Comparison for IBS.

When only incorporating clinical data and gene expression, the two most informative modalities, we found that all methods that incorporated the group structure of the data into their model-building process performed similarly in terms of discrimination, as measured by Antolini’s C (Table 1), except *Late (MoE)*, which underperformed the other approaches. *BlockForest* achieved the highest Antolini’s C, but most other methods did not perform significantly worse than it (Table 1). All methods that disregarded group structure (*Early*, Elastic net, and RSF) performed significantly worse than *BlockForest* in terms of Antolini’s C. In terms of model IBS, *BlockForest* significantly outperformed all other methods. In addition, we saw a clear trend of deep learning models underperforming compared to statistical ones in terms of IBS, suggesting that deep learning models tend to be calibrated worse than statistical ones, even when using the same loss function. In fact, the best deep learning models performed worse in terms of IBS than the worst statistical ones (Table 1). When looking beyond aggregated performance measures and investigating the rank of each model on each cancer-type-specific dataset in terms of Antolini’s C and the IBS we found a similar picture, with *BlockForest* and some of the intermediate deep learning methods generally securing the higher ranks for Antolini’s C, while *BlockForest* and other statistical models dominated in terms of the IBS (Figures 1A and 1B).

**Table 1:** All considered methods showed a lack of noise resistance, exemplified by their inability to outperform the clinical baselines

	Antolini’s C^a^		IBS^b^	
	Clinical + GEX	All	Clinical + GEX	All
BlockForest	0.637 (0.0059)**^∗,^**^c^	0.619 (0.0060)**^∗^**	0.162 (0.0020)**^∗,^**^c^^,^^d^	0.164 (0.0021)**^∗,^**^d^
Elastic net	0.599 (0.0061)^c^	0.589 (0.0060)	0.167 (0.0021)	0.168 (0.0021)
PriorityLasso	0.632 (0.0060)**^∗,^**^c^	0.626 (0.0063)**^∗^**	0.166 (0.0022)^c^	0.174 (0.0024)
RSF	0.601 (0.0055)^c^	0.575 (0.0059)	0.167 (0.0020)^c^	0.169 (0.0021)
Early	0.607 (0.0058)^c^	0.587 (0.0059)	0.176 (0.0025)^c^	0.179 (0.0025)
Intermediate (Attention)	0.633 (0.0057)**^∗,^**^c^	0.616 (0.0055)	0.173 (0.0026)	0.177 (0.0026)
Intermediate (Concat)	0.635 (0.0057)**^∗,^**^c^	0.624 (0.0053)**^∗^**	0.173 (0.0026)^c^	0.190 (0.0035)
Intermediate (Embrace)	0.622 (0.0056)**^∗,^**^c^	0.575 (0.0057)	0.172 (0.0024)^c^	0.182 (0.0026)
Intermediate (Max)	0.635 (0.0055)**^∗,^**^c^	0.602 (0.0056)	0.174 (0.0025)^c^	0.186 (0.0027)
Intermediate (Mean)	0.636 (0.0054)**^∗,^**^c^	0.615 (0.0056)	0.171 (0.0023)^c^	0.176 (0.0025)
Late (Mean)	0.631 (0.0056)**^∗^**	0.627 (0.0054)**^∗^**	0.172 (0.0025)	0.171 (0.0023)
Late (MoE)	0.599 (0.0056)^c^	0.564 (0.0054)	0.179 (0.0024)^c^	0.188 (0.0034)

Mean Antolini’s C and IBS across test splits and datasets when integrating clinical data and gene expression compared to integrating all of the available modalities (described in Table S1). Standard errors are in parentheses. ^∗^ indicates values that were not significantly worse than the best model per column.

a denotes a metric for which higher values are better.

b the opposite.

c indicates significantly better performance for a model utilizing clinical data and gene expression compared to all available modalities. P values are based on paired one-sided Wilcoxon signed-rank tests, except for the unimodal comparison, which is based on two-sided paired Wilcoxon signed-rank tests (see STAR Methods for details).

d indicates significantly better performance than the best clinical-only model.

None of the considered models trained on clinical data and gene expression were able to significantly outperform the best unimodal method in terms of Antolini’s C (a neural network trained on only clinical data), and only *BlockForest* outperformed the best unimodal method in terms of the IBS (an RSF trained only on clinical data) (Table 1).

### Existing multi-omics integration survival methods exhibit a lack of noise resistance

As a first step to gauge the noise resistance of each model, we considered the scenario of fitting all methods on all seven available modalities (Table S1). Because clinical data (usually) contain much predictive information and yet are very low-dimensional, including additional high-dimensional modalities may “crowd out” some of the predictiveness of the clinical data.^12^ Published work has found decreases in performance when integrating all available modalities.^13^^,^^21^^,^^26^ Thus, in the first part of our second experiment, our goal was to verify (1) whether this crowding-out would be visible across integration methods and (2) which method could handle the crowding-out the best (if present).

All of our considered models except *Late (Mean)* performed significantly worse in terms of Antolini’s C when integrating all available modalities compared with clinical data and gene expression (Table 1). Similarly, all models except *Intermediate (Attention)*, *Late (Mean)*, and the Elastic net performed significantly worse in terms of the IBS when integrating all available modalities, relative to integrating only clinical data and gene expression (Table 1). For Antolini’s C, most methods decreased by similar amounts (usually between 0.01 and 0.02, with *Late (MoE)* being the outlier with a decrease of 0.035). Comparing the rank by cancer-type-specific dataset of each integration method in terms of Antolini’s C and the IBS told a similar story, with most methods performing better when integrating clinical data and gene expression compared to all modalities on all but a handful of datasets (Figures 1C and 1D).

**Figure 2:**
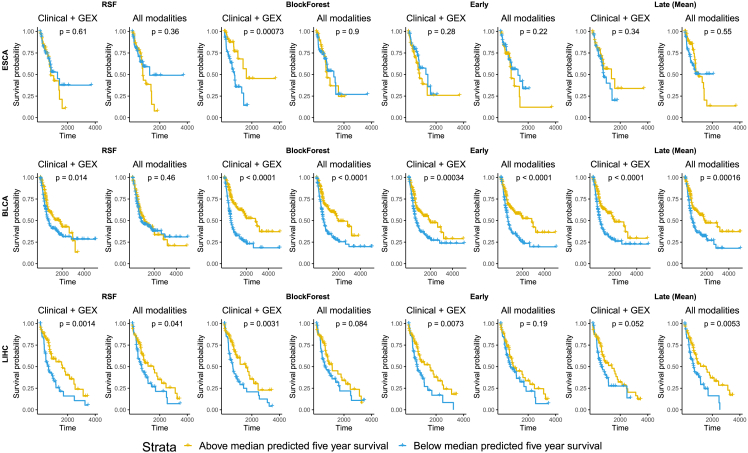
Including more modalities beyond clinical data and gene expression often reduced the patient stratification ability of the considered methods as measured by the p value of the log rank test Kaplan-Meier curves stratified by whether a patient was below or above the median predicted 5-year survival according to the model in question (see STAR Methods for further details). RSF, *BlockForest*, *Early*, and *Late (Mean)* shown exemplarity on esophageal (ESCA), head, neck, and shoulder (HNSC), and lung (LUSC) cancers, see supplemental information for all datasets and additional models (Figures S1 and S2). P values are based on log rank tests.

Methods that did not use the group structure of the multi-omics data (e.g., RSF and *Early*) also decreased their stratification ability when integrating additional, less informative modalities. In particular, the log rank test between two Kaplan-Meier (KM) curves, created by stratifying on the median predicted patient-level risk, commonly went from significant when integrating clinical data and gene expression to non-significant when integrating all available modalities (Figure 2 and Figures S1 and S2) (see STAR Methods for details on KM curves). Group-aware methods such as *BlockForest* or *Late (Mean)* were more stable in terms of stratification (mirroring our Antolini’s C results), but even these sometimes suffered significantly in terms of stratification ability when integrating less informative modalities (e.g., *BlockForest* on esophageal cancer [ESCA]).

Thus, we found that practically all considered integration methods were affected by a lack of noise resistance. Furthermore, as mentioned previously, none of the methods were able to outperform the best unimodal baseline for both considered metrics (and only one model, *BlockForest*, outperformed the best unimodal method in terms of IBS), even when “only” integrating clinical data and gene expression. Therefore, all tested methods seemed unable to properly weigh the importance of each of the modalities in question.

Some methods we considered stood out as performing better than others in terms of both noise resistance and overall performance. Among the considered deep learning integration methods, *Late (Mean)* was the overall best performer. *Late (Mean)* performed well in terms of Antolini’s C and did not perform significantly worse when trained on clinical data and gene expression compared to using all modalities. Among statistical methods, both *BlockForest* and *PriorityLasso* methods showed strong overall performance, with *BlockForest* performing particularly well on clinical data and gene expression. However, *BlockForest* experienced a sharp drop in discriminative performance when integrating all modalities (compared to only clinical data and gene expression), while *PriorityLasso* exhibited relatively better noise resistance.

To control for the possibility of the dimensionality of each modality playing a large role in our results, we performed an auxiliary experiment. Concretely, we executed the same experiment as before but performed an initial PCA step per input modality (except clinical, since it was already low-dimensional) before training each model. We found that performing the initial PCA step significantly improved performance for many of the considered methods (Table 2), though their noise resistance was generally unaffected, except for methods disregarding the group structure. For *BlockForest*, an initial PCA step increased noise resistance, leading to similar Antolini’s C whether integrating all modalities or only gene expression and clinical data.

**Table 2:** Dimensionality reduction improved the performance of most methods but did not generally lead to superior noise resistance

	Antolini’s concordance				IBS			
	Clinical + GEX		All		Clinical + GEX		All	
	32 PCs	64 PCs	32 PCs	64 PCs	32 PCs	64 PCs	32 PCs	64 PCs
BlockForest	0.635 (0.0060)	0.635 (0.0061)	0.635 (0.0062)^∗^	0.638 (0.0060)^∗^	0.164 (0.0020)	0.165 (0.0020)	0.164 (0.0020)	0.165 (0.0020)
Elastic net	0.636 (0.0059)^∗^	0.625 (0.0060)^∗^	0.613 (0.0061)^∗^	0.606 (0.0059)^∗^	0.164 (0.0021)^∗^	0.166 (0.0021)^∗^	0.166 (0.0021)^∗^	0.167 (0.0021)
PriorityLasso	0.641 (0.0059)^∗^	0.635 (0.0060)	0.636 (0.0059)	0.634 (0.0058)	0.165 (0.0021)	0.165 (0.0021)	0.170 (0.0023)^∗^	0.170 (0.0023)^∗^
RSF	0.629 (0.0057)^∗^	0.623 (0.0059)^∗^	0.617 (0.0057)^∗^	0.609 (0.0058)^∗^	0.166 (0.0019)^∗^	0.168 (0.0019)	0.167 (0.0020)	0.169 (0.0020)
Early	0.606 (0.0057)	0.602 (0.0058)	0.595 (0.0057)	0.591 (0.0058)	0.173 (0.0023)^∗^	0.176 (0.0024)	0.177 (0.0025)	0.180 (0.0026)
Intermediate (Attention)	0.629 (0.0059)	0.629 (0.0057)	0.607 (0.0060)	0.612 (0.0057)	0.168 (0.0023)^∗^	0.170 (0.0023)	0.173 (0.0024)	0.175 (0.0024)
Intermediate (Concat)	0.631 (0.0057)	0.632 (0.0059)	0.628 (0.0055)	0.624 (0.0056)	0.167 (0.0023)^∗^	0.170 (0.0024)^∗^	0.179 (0.0027)	0.183 (0.0028)
Intermediate (Embrace)	0.625 (0.0060)	0.634 (0.0058)^∗^	0.575 (0.0060)	0.581 (0.0062)	0.168 (0.0022)^∗^	0.167 (0.0022)^∗^	0.172 (0.0022)^∗^	0.171 (0.0021)
Intermediate (Max)	0.629 (0.0058)	0.627 (0.0058)	0.597 (0.0057)	0.600 (0.0058)	0.168 (0.0023)^∗^	0.169 (0.0023)^∗^	0.176 (0.0024)^∗^	0.177 (0.0025)^∗^
Intermediate (Mean)	0.636 (0.0059)	0.635 (0.0056)	0.614 (0.0056)	0.614 (0.0057)	0.167 (0.0023)^∗^	0.169 (0.0023)	0.173 (0.0023)	0.175 (0.0024)^∗^
Late (Mean)	0.633 (0.0058)	0.628 (0.0059)	0.622 (0.0057)	0.626 (0.0055)	0.168 (0.0022)^∗^	0.170 (0.0023)^∗^	0.169 (0.0023)^∗^	0.171 (0.0023)
Late (MoE)	0.597 (0.0057)	0.597 (0.0058)	0.561 (0.0054)	0.566 (0.0053)	0.175 (0.0022)^∗^	0.174 (0.0023)^∗^	0.178 (0.0023)^∗^	0.177 (0.0023)^∗^

Mean Antolini’s C and IBS across test splits of the considered datasets on TCGA when integrating clinical data and gene expression or all available modalities combined with using PCA for dimensionality reduction per modality, with a specific number of components. Standard errors are in parentheses. ∗ indicates significantly better performance compared to the same model trained without dimensionality reduction (Table 1). The number of principal components (PCs) k indicates that PCA was performed using k principal components. ↑ denotes a metric for which higher values are better, ↓ the opposite. P-values are based on paired one-sided Wilcoxon signed-rank tests (see STAR Methods for details).

We also considered the application of a multimodal dropout unit (see STAR Methods for details). The usage of the multimodal dropout unit significantly improved discriminative performance for two of the models (Table S4). Despite this, even with multimodal dropout, the considered deep learning methods performed worse with clinical data and gene expression compared with integrating all available modalities.

Next, we evaluated the performance of different methods on simulated data that included simulated modalities representing Gaussian noise (as described in “research questions and matching experiments” and the STAR Methods). Our simulations led to similar conclusions as the experiment of integrating multiple modalities. All methods declined in performance as more noise modalities were included as inputs (Table 3). However, some methods were more resistant to noise than others, with the group-naive methods (*Early*, Elastic net, and RSF) showing the least decline in both Antolini’s C and IBS. Some group-aware methods, such as *PriorityLasso*, were also relatively resistant to noise. When integrating one noise modality, *Intermediate (Embrace)*, *Late (MoE)*, and RSF performed significantly worse in terms of Antolini’s C compared to using only clinical data and gene expression. However, when integrating five noise modalities, all methods except RSF, *PriorityLasso*, and *Early* performed significantly worse than the same model when integrating clinical data and gene expression. A similar trend was observed for model calibration as (partially) measured by the IBS, with all methods except RSF performing significantly worse than clinical data and gene expression when integrating five noise modalities.

**Table 3:** Most methods decreased in performance significantly when multiple Gaussian noise modalities were used in training, relative to training only on clinical data and gene expression

	Antolini’s concordance			Integrated Brier Score		
	1 noise modality	3 noise modalities	5 noise modalities	1 noise modality	3 noise modalities	5 noise modalities
BlockForest	0.641 (0.0059)	0.631 (0.0064)	0.627 (0.0062)^†^	0.164 (0.0020)^†^	0.165 (0.0020)^†^	0.166 (0.0020)^†^
Elastic net	0.595 (0.0063)	0.593 (0.0060)	0.589 (0.0063)^†^	0.167 (0.0021)	0.167 (0.0021)	0.167 (0.0021)
PriorityLasso	0.631 (0.0061)	0.624 (0.0063)^†^	0.624 (0.0064)	0.166 (0.0022)	0.168 (0.0022)^†^	0.169 (0.0023)^†^
RSF	0.598 (0.0057)^†^	0.595 (0.0056)^†^	0.599 (0.0055)	0.167 (0.0020)	0.168 (0.0020)	0.168 (0.0020)
Early	0.606 (0.0055)	0.608 (0.0057)	0.603 (0.0057)	0.177 (0.0025)	0.178 (0.0024)	0.179 (0.0025)^†^
Intermediate (Attention)	0.630 (0.0059)	0.620 (0.0055)^†^	0.613 (0.0056)^†^	0.175 (0.0024)	0.185 (0.0024)^†^	0.189 (0.0022)^†^
Intermediate (Concat)	0.628 (0.0057)	0.625 (0.0058)^†^	0.619 (0.0060)^†^	0.186 (0.0026)^†^	0.201 (0.0032)^†^	0.213 (0.0037)^†^
Intermediate (Embrace)	0.609 (0.0055)^†^	0.585 (0.0055)^†^	0.575 (0.0057)^†^	0.175 (0.0024)^†^	0.183 (0.0024)^†^	0.185 (0.0023)^†^
Intermediate (Max)	0.635 (0.0056)	0.626 (0.0054)^†^	0.618 (0.0057)^†^	0.178 (0.0025)^†^	0.189 (0.0027)^†^	0.198 (0.0028)^†^
Intermediate (Mean)	0.629 (0.0057)	0.614 (0.0058)^†^	0.614 (0.0058)^†^	0.176 (0.0025)^†^	0.184 (0.0024)^†^	0.188 (0.0024)^†^
Late (Mean)	0.629 (0.0055)	0.625 (0.0057)	0.615 (0.0056)^†^	0.176 (0.0023)^†^	0.185 (0.0023)^†^	0.195 (0.0027)^†^
Late (MoE)	0.573 (0.0049)^†^	0.546 (0.0048)^†^	0.541 (0.0049)^†^	0.186 (0.0024)^†^	0.196 (0.0032)^†^	0.204 (0.0034)^†^

Mean Antolini’s C and IBS across test splits and datasets when integrating clinical data and gene expression along with up to five simulated noise modalities, and optionally, the true survival time (see STAR Methods). Standard errors are in parentheses. k noise modalities indicates that the model was trained with k noise modalities of dimensionality 10,000, in addition to clinical data and gene expression. † indicates performance significantly worse than the same model trained on only clinical data and gene expression (without noise modalities and target). ↑ denotes a metric for which higher values are better, ↓ the opposite. P values are based on paired one-sided Wilcoxon signed-rank tests (see STAR Methods for details).

Last, to better understand how different integration methods behaved in the presence of very informative but low-dimensional modalities, we moved on to the simulations containing one strongly informative modality in addition to the previously considered modalities simulating pure noise.

### Statistical methods effectively leverage highly informative modalities

Including true survival times in the covariates showed a drastic inability of most methods to use the information in low-dimensional but highly predictive modalities. In particular, when we allowed for the model access to observed survival times, as a proxy of an extremely predictive but very low-dimensional modality (since survival time was added to the clinical modality), all models except RSF, *Late (MoE)*, and *Early* drastically improved their performance (Table 4). Methods ignoring the group structure did not benefit as much as some of the other methods from the highly informative modality for the same reason that some of them did not decay as much in performance in previous noisy simulations; group-naive methods tended to ignore low-dimensional modalities. Interestingly, the Elastic net also greatly improved its performance, despite ignoring the group structure, presumably due to its ability to perform feature selection.

**Table 4:** Including survival times in the clinical modality greatly improved performance for all methods, but most methods were not able to maintain this gain when integrating additional Gaussian noise modalities

	Antolini’s concordance			
	0 noise modalities	1 noise modality	3 noise modalities	5 noise modalities
BlockForest	0.894 (0.0039)^∗^	0.907 (0.0036)^∗^	0.912 (0.0041)^∗^	0.914 (0.0043)^∗^
Elastic net	0.923 (0.0034)^∗^	0.916 (0.0037)^∗^	0.912 (0.0038)^∗^	0.905 (0.0047)^∗^
PriorityLasso	0.955 (0.0056)^∗^	0.952 (0.0056)^∗^	0.951 (0.0057)^∗^	0.950 (0.0056)^∗^
RSF	0.609 (0.0055)^∗^	0.610 (0.0055)^∗^	0.610 (0.0055)^∗^	0.607 (0.0055)^∗^
Early	0.609 (0.0057)	0.604 (0.0058)	0.606 (0.0055)	0.607 (0.0056)
Intermediate (Attention)	0.847 (0.0055)^∗^	0.818 (0.0056)^∗^	0.766 (0.0068)^∗^	0.730 (0.0074)^∗^
Intermediate (Concat)	0.865 (0.0055)^∗^	0.850 (0.0058)^∗^	0.827 (0.0065)^∗^	0.816 (0.0063)^∗^
Intermediate (Embrace)	0.809 (0.0056)^∗^	0.743 (0.0060)^∗^	0.672 (0.0064)^∗^	0.639 (0.0062)
Intermediate (Max)	0.841 (0.0052)^∗^	0.804 (0.0059)^∗^	0.779 (0.0060)^∗^	0.762 (0.0061)^∗^
Intermediate (Mean)	0.851 (0.0052)^∗^	0.816 (0.0056)^∗^	0.770 (0.0064)^∗^	0.740 (0.0067)^∗^
Late (Mean)	0.853 (0.0054)^∗^	0.837 (0.0057)^∗^	0.800 (0.0071)^∗^	0.773 (0.0076)^∗^
Late (MoE)	0.691 (0.0053)^∗^	0.634 (0.0056)^∗^	0.579 (0.0051)	0.554 (0.0047)

Mean Antolini’s C and IBS across test splits and datasets when integrating clinical data and gene expression along with up to five simulated noise modalities and survival times as part of the clinical modality (see STAR Methods). Standard errors are in parentheses. k noise modalities indicates that the model was trained with k noise modalities of dimensionality 10,000, in addition to clinical data and gene expression. “With target” indicates that the true survival time was added to the clinical features. ∗ indicates performance significantly better than the same model trained on only clinical data and gene expression (without noise modalities and target). ↑ denotes a metric for which higher values are better, ↓ the opposite. P values are based on paired one-sided Wilcoxon signed-rank tests (see STAR Methods for details).

Although all other methods drastically improved their performance initially, most of them very quickly decreased their performance again once additional pure-noise modalities were added, suggesting that these methods will struggle when not every modality contains predictive information. We found this was especially the case for the deep learning methods, all of which decreased in Antolini’s C considerably when adding five as opposed to one noise modality while including a highly informative modality based on the survival time (Table 4). The statistical group-aware methods, *PriorityLasso* and *BlockForest* were both quite stable and achieved virtually the same (or even slightly better) discriminative performance whether one, three, or five noise modalities were added in addition to the observed survival times.

## Discussion

In our work, we performed a large-scale empirical comparison of different integration methods for multi-omics survival methods. Chiefly, we were interested in the noise resistance of these methods. In the scope of our first research question, we investigated which multi-omics integration method performed best in a low-noise setting, where all modalities to be integrated provided high predictive value. *BlockForest*, a group-aware RSF variant, performed the best when the models were set to integrate only clinical data and gene expression. Otherwise, late fusion approaches dominated, with *PriorityLasso*, a linear sequential late fusion approach, and *Late (Mean)* being among the best performers in terms of both discriminative and calibration performance. Sophisticated intermediate neural fusion techniques such as attention or the Embrace network were not able to outperform comparatively simple choices such as max-pooling or concatenation, matching the results of Vale-Silva and Rohr.^21^ Early fusion approaches (that is, effectively ignoring the group structure) underperformed all other integration methods and we thus caution against using them, even when integrating highly informative modalities, because low-dimensional modalities such as clinical data were crowded out more easily by higher dimensional modalities, even when the total number of modalities was small.

Second, our comparison dealt with the key challenge of which multi-omics integration method performed best in noisy settings. Given that we found all considered integration methods to decrease their performance in noisy settings (Tables 1 and 3), we caution against using multi-omics survival integration methods in settings in which the informativeness of the modalities is unknown. Thus, until novel methods with stronger noise-resistant properties are developed, we recommend relying on only modalities that are known to have high predictive value for patient survival, such as clinical data and potentially gene expression.

Last, our experiments addressed the question of which multi-omics integration methods were best able to leverage highly informative modalities despite the presence of noisy modalities. We found that *PriorityLasso* proved best in such settings. Although our simple simulated example may seem contrived, it has direct applications. New modalities, for which it is not well-understood how predictive they are for a particular cancer type, may become available soon. In these cases, applying a model which is resistant to noise yet can account for low-dimensional but highly predictive modalities is paramount.

Overall, our empirical comparison revealed and confirmed several important facts. First, the most commonly considered multimodal integration methods for multi-omics survival analysis are not sufficiently noise resistant. That is, as more modalities get added beyond the most predictive ones, performance decreases, sometimes drastically so. Further, in optimal settings, late fusion can be a promising technique for multi-omics integration, despite a comparative lack of research on it in the (deep learning) multi-omics survival community relative to, for example, intermediate fusion. Last, although none of the current methods proved close to ideal multi-omics integration, *PriorityLasso* stood out in terms of overall performance, interpretability, and noise resistance. Thus, if a survival prediction from multi-omics data is desired and the informativeness of modalities is not clear, we recommend using *PriorityLasso*.

**Figure 3:**
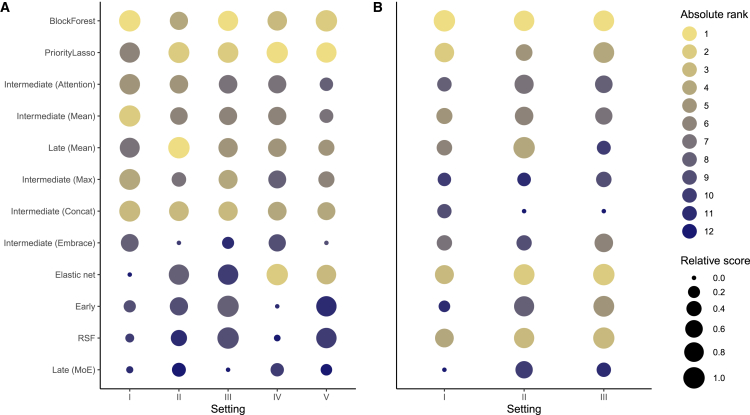
*PriorityLasso*, *BlockForest*, and *Late (Mean)* stood out as the best performers in our empirical comparison Bubble plot summarizing the absolute and relative performance of all methods. The relative score denotes the delta to the same (or similar) model type in an easier setting (see settings I–V for details), while the absolute rank compares all models to each other in the same setting. (A) Antolini’s concordance (Antolini’s C). (B) Integrated Brier Score (IBS). Settings: I. Clinical + GEX (relative score comparison: best clinical-only model). II. All modalities (relative score comparison: same model when trained on Clinical + GEX). III. Clinical + GEX + five noise modalities (relative score comparison: same model when trained on Clinical + GEX). IV. Clinical + GEX + survival times (relative score comparison: same model when trained on Clinical + GEX). V. Clinical + GEX + survival times + five noise modalities (relative score comparison: same model when trained on Clinical + GEX + survival times). All relative scores were min-max normalized.

Overall, *PriorityLasso* and *BlockForest* stood out as the best methods in virtually all considered settings, especially in terms of calibration, where all deep learning methods underperformed relative to statistical methods (Figure 3). We hope that Figure 3 may guide researchers in choosing the right model for their application, depending on their needs.

### Limitations of the study

It is worth noting that our work has certain limitations. Since we focus on datasets from TCGA, it cannot be guaranteed to generalize to other input datasets—for example, due to a difference in the informativeness of the clinical data modality. In fact, the generalization of the present work to additional datasets and projects, such as METABRIC^28^ and TARGET would certainly represent an interesting extension. Except for RSF and *BlockForest*, we restricted ourselves to methods based on the Cox PH model. Although we consider it unlikely, it may be that other survival loss functions, such as the Deep Extended Hazard model^29^ and others might have exhibited improved noise resistance. In addition, due to the extremely large space of possible preprocessing options, we were not able to consider all possibilities with respect to preprocessing. In particular, while some works consider gene filtering, we instead tested each model’s ability to handle high-dimensional modalities.

There are several key areas for potential future work. One possible future direction is increasing the noise resistance of multi-omics survival analysis models. In particular, meta-learning approaches may prove fruitful to learning modality importance across cancers. Alternatively, automatic (that is, jointly with the coefficients) learning of the optimal modality order for sequential learning approaches such as *PriorityLasso* would also be a worthwhile goal.

## STAR★Methods

### Key resources table

**Table:** 

REAGENT or RESOURCE	SOURCE	IDENTIFIER
**Deposited data**		

TCGA PANCANATLAS data (RNA (Final), RPPA (Final), DNA Methylation (Merged 27K+450K Only), miRNA (Batch Effects Normalized miRNA data), Mutations, TCGA-Clinical Data Resource (CDR) Outcome)	National Cancer Institute	RRID:SCR_003193; https://gdc.cancer.gov/about-data/publications/pancanatlas
Xenabrowser PANCAN GISTIC2 thresholded data	University of Santa Cruz	RRID:SCR_018938; https://xenabrowser.net/datapages/?cohort=TCGA%20Pan-Cancer%20(PANCAN)&removeHub=https%3A%2F%2Fxena.treehouse.gi.ucsc.edu%3A443

**Software and algorithms**		

Data and code generated for this study	This paper	Zenodo: https://doi.org/10.5281/zenodo.7529459; GitHub: https://github.com/BoevaLab/Multi-omics-noise-resistance
Python 3.10	The Python Software Foundation	https://www.python.org/; RRID:SCR_008394
R 4.1.3	The R Project for Statistical Computing	https://www.r-project.org/; RRID:SCR_001905
Numpy 1.23.0	The NumPy community	https://numpy.org/; RRID:SCR_008633
scikit-learn 1.1.1	The scikit-learn community	https://scikit-learn.org/; RRID:SCR_002577
Pandas 1.4.3	The Pandas community	RRID:SCR_018214; https://pandas.pydata.org/
Skorch 0.12.0	The skorch community	https://skorch.readthedocs.io/en/stable/
Pytorch 1.12.0	The Linux Foundation	RRID:SCR_018536; https://pytorch.org/
scikit-survival 0.19.0	The scikit-survival community	https://scikit-survival.readthedocs.io/en/stable/
mlr3 0.13.3	The mlr3 community	https://mlr3.mlr-org.com/
mlr3proba 0.4.9	The mlr3proba community	https://mlr3proba.mlr-org.com/
glmnet 4.1.4	The glmnet community	https://glmnet.stanford.edu/articles/glmnet.html
prioritylasso 0.2.5	The prioritylasso community	https://cran.r-project.org/web/packages/prioritylasso/index.html
blockForest 0.2.4	The blockForest community	https://cran.r-project.org/web/packages/blockForest/index.html
ranger 0.13.1	The ranger community	https://cran.r-project.org/web/packages/ranger/index.html
ggplot2 3.3.6	The ggplot2 community	RRID:SCR_014601; https://ggplot2.tidyverse.org/

### Resource availability

#### Lead contact

Further information and requests for resources and reagents should be directed to and will be fulfilled by the lead contact, Valentina Boeva (valentina.boeva@inf.ethz.ch).

#### Materials availability

This study did not generate new unique reagents.

### Method details

#### Datasets

In our study, we considered 17 datasets from The Cancer Genome Atlas (TCGA).^30^ Specifically, we chose only datasets that had available all modalities of interest, namely, clinical data, gene expression, protein expression, Copy Number Variation (CNV), methylation, mutation, and micro-RNA (Table S1). We preprocessed data as in Wissel et al.^31^

Briefly, we preprocessed all data directly from the *PANCANATLAS*,^30^ except for CNV, which we took from *Xenabrowser*.^32^ We used overall survival (OS) as the endpoint as per Liu et al.^33^ Missing numerical variables that were missing for more than 10% of samples were removed, while those missing for less than 10% were imputed using the median across non-missing samples. Missing categorical variables were assigned a separate category. All expression data were log-transformed. For clinical features, we chose a common set across all cancer types, namely age at diagnosis, gender, race, tumor stage, clinical stage, and histological type, when available. Clinical variables that were either not available or not applicable for a particular cancer type were excluded for that cancer type. For further details on data preprocessing, we refer to Wissel et al.^31^

#### Deep learning multimodal integration methods

Our work distinguished between early fusion, intermediate fusion, and late fusion.^17^^,^^18^ Let $X\in R^{n\times p}$ denote the design matrix with n patients each having p covariates. Let $\left\{m_{1},\ldots ,m_{M}\right\}$ denote the set of modalities where M is the total number of considered modalities. Let $X^{\left(m_{i}\right)}$ denote the design matrix containing only covariates from the i-th modality.

*Early fusion* concatenates all modalities at the start and fits a joint, fully-connected neural network over all features to estimate the log hazard θ: $\theta =g\left(F_{\text{early}}\left(X^{\left(m_{1}\right)},\ldots ,X^{\left(m_{M}\right)}\right)\right)$, where g denotes a fully connected joint neural network, F denotes a fusion method and $F_{\text{early}}\left(X^{\left(m_{1}\right)},\ldots ,X^{\left(m_{M}\right)}\right)$ performs concatenation of all features without any transformation.

*Intermediate fusion* first fits modality-specific subnetworks $h_{1},\ldots ,h_{M}$, where $h_{i}:R^{n\times m_{i}}\to R^{n\times q}$, before performing an aggregation $F_{\text{intermediate}}:R^{n\times Mq}\to R^{n\times k}$, where generally k≤Mq. Afterward, the aggregated representation output by $F_{\text{intermediate}}$ is used as an input into a joint network to produce a prediction of the log hazard: $\theta =g\left(F_{\text{intermediate}}\left(h_{1}\left(X^{\left(m_{1}\right)}\right),\ldots ,h_{M}\left(X^{\left(m_{M}\right)}\right)\right)\right)$.

Lastly, *late fusion* learns modality-specific log hazards $f_{1},\ldots ,f_{M}$, where $f_{i}\in R^{n}$, which are aggregated using a weighting matrix $W\in R^{n\times M}$: $\theta =\sum_{i=1}^{M}W_{i}f_{i}\left(X^{\left(m_{i}\right)}\right)$.

Below, we provide details on the early, intermediate, and late fusion methods we included in our benchmark.

**∗**Early fusion Since early fusion merely concatenates all features before fitting a joint network, we only considered this variant, which we referred to it as ***Early*** throughout our experiments.
**∗**Intermediate fusion For intermediate fusion, we considered the following five methods.

##### Mean-pooling

$F_{\text{mean}}=\frac{1}{M}\sum_{i=1}^{M}g_{i}\left(X^{\left(m_{i}\right)}\right)\in R^{n\times q}$. We referred to this variant as ***Intermediate (Mean)***.

##### Max-pooling

$F_{\max}=\max\left(g_{1}\left(X^{\left(m_{1}\right)}\right),\ldots ,g_{M}\left(X^{\left(m_{M}\right)}\right)\right)\in R^{n\times q}$, where max denotes an element-wise maximum. Throughout the text, we deemed this method ***Intermediate (Max)***.

##### Concatenation

$F_{\text{concat}}=\left[g_{1}\left(X^{m_{1}}\right),\ldots ,g_{M}\left(X^{\left(m_{M}\right)}\right)\right]\in R^{n\times Mq}$. This variant was denoted ***Intermediate (Concat)***.

##### Attention

$F_{\text{attention}}=\sum_{i=1}^{M}A_{i}g_{i}\left(X^{\left(m_{i}\right)}\right)\in R^{n\times q}$, where $A\in R^{n\times M}$ is a matrix of learned attention weights. We called this fusion method ***Intermediate (Attention)***.

##### Embrace

The Embrace approach is multimodal fusion based on multinomial sampling, effectively sampling one modality for each element of the fused feature vector, for each patient. Thus, $F_{\text{Embrace}}\left(g_{1}\left(X^{m_{1}}\right),\ldots ,g_{M}\left(X^{m_{M}}\right)\right)\in R^{n\times q}$.^34^ We refer to Choi and Lee^34^ for full details for brevity. This last variant of intermediate fusion was denoted as ***Intermediate (Embrace)***.

**∗**Late fusion Two strategies were considered to represent the family of late fusion methods.

##### Late mean-fusion

$W_{ij}=\frac{1}{M}\forall i\in \left\{1,\ldots ,n\right\}\forall j\in \left\{1,\ldots ,M\right\}$. Later, we referred to this fusion method as ***Late (Mean)***.

##### Mixture of Experts (MoE)

W is learned using a linear gating network taking in the complete data X, followed by a softmax activation. In our benchmarks, we denoted this method **Late (Mixture of Experts (MoE))**.

We note that while we cannot possibly include all previous integration techniques in our study, our current work subsumes most that we are aware of. For example, our considered methods implicitly included the work of Cheerla and Gevaert^19^ (a neural network with intermediate mean fusion trained using the Cox PH loss) and partly the methods proposed in Tong et al.^24^ (a modality-specific autoencoder that is combined using intermediate fusion with concatenation).

#### Neural network training details

We developed all neural networks using PyTorch^35^ and skorch.^36^ We used batch normalization throughout the networks^37^ and Rectified Linear Units for non-linear activations.^38^ For all neural networks, we performed standardization of all features before fitting the model based on the training set. Categorical variables were one-hot encoded; new categorical levels first appearing in the test set had all of their categories set to zero. All fusion methods were also implemented in PyTorch. For Embrace fusion and attention-based fusion, we used the implementations of Vale-Silva and Rohr.^21^ All integration methods were fit with two hidden layers, 128 nodes within the first hidden layer and 64 nodes within the second hidden layer. All layers except the final linear layer outputting the log hazard were fit with biases. Zero-variance features were removed for all deep learning models at training time.

The training was performed using a validation set of 10% of the training set which was used for both early stopping and learning rate scheduling. We fitted all models using the *Adam* optimizer^39^ with a default initial learning rate of 0.01 which was reduced by a factor of 10 if the validation loss did not decrease for five epochs. We trained all models for a maximum of 100 epochs using no batching since all datasets were quite small (Table S1) and performed early stopping with patience of 10 using the validation loss. All networks were trained using the negative partial log likelihood with the Breslow approximation for ties.^27^ Survival functions were calculated using the Breslow estimator implementation from scikit-survival.^27^^,^^40^

We performed 5-fold cross-validation, stratified on the event indicator, to find the optimal hyperparameters for both the dropout probability and the weight decay hyperparameter for *Adam*. In particular, we selected the dropout probability from {0.0,0.25,0.5} and the weight decay from {0.1,0.01,0.001} based on which combination achieved the highest log likelihood across validation folds using grid search stratified by the event indicator. Grid search was implemented using GridSearchCV from scikit-learn.

#### Multimodal dropout

Some models were trained using multimodal dropout (Table S4). Multimodal dropout^19^^,^^21^ functions identical to ordinary input dropout units, except on the modality as opposed to the covariate level. We implemented multimodal dropout in Pytorch using inverted dropout. The probability for multimodal dropout was tuned by adding it to the grid along with the other hyperparameters and selecting it from {0.0,0.25,0.5}.

#### Statistical multimodal integration methods

We included four statistical methods in our comparisons, two of which used the group structure of the multi-omics data and two of which did not. All statistical methods were benchmarked using mlr3^41^ and mlr3proba.^42^ Zero-variance features were removed for all statistical models at training time.

##### Random survival forest (RSF)^3^

We implemented the Random Survival Forest using ranger.^43^ Specifically, we trained 2000 trees and used the *extratrees* split rule (splitrule = ”extratrees”). All other hyperparameters of ranger were left at the default.

We passed categorical covariates to ranger as factors and did not standardize numerical covariates.

##### BlockForest^13^

Since *BlockForest* uses ranger internally, we describe only the settings that are specific to *BlockForest*, all others were set equivalently to RSF.

We trained *BlockForest* using the blockForest package (block.method = ”BlockForest”). We further used 300 sets of randomly generated hyperparameter modality weight candidates and 100 trees in each forest during tuning, to decrease computation time.

##### Elastic net

We used glmnet^44^^,^^45^ to implement the Elastic net with the Breslow^27^ approximation for ties. The regularization hyperparameter was chosen using 5-fold cross-validation with grouped = TRUE, based on which gave the highest log likelihood across validation folds (type.measure = ”deviance” and lambda.type = ”min”). We used the default glmnet path for the regularization hyperparameter. The ridge fraction of the Elastic net was fixed at 0.05 to remedy the issues commonly encountered by the Lasso for very high correlations (alpha = 0.95). Categorical covariates were dummy encoded and all covariates were standardized using glmnet (standardize=TRUE). Categorical levels first appearing in the test set had all of their categories set to zero.

##### PriorityLasso^16^

The priority order for *PriorityLasso* was chosen using an initial Ridge step as described in Herrmann et al.^12^ The offsets were estimated using 5-fold cross-validation (cvoffset = TRUE and cvoffsetnfolds = 5).

Since *PriorityLasso* uses glmnet internally, all other hyperparameters for it were set equivalently to the Elastic net hyperparameters.

#### Principal component analysis (PCA)

Each PCA for the experiments in question (Table 2) was calculated on the training set using either scikit-learn (for the deep learning methods) or mlr3 (for the statistical methods). The test set was projected onto the principal components learned on the training set. All covariates were standardized before fitting each PCA.

#### Kaplan-Meier (KM) curves

To visually highlight differences between models when integrating only informative modalities compared to all modalities, we computed KM curves for good and bad prognosis patients as predicted by each model †(Figures 2, S1, and S2). First, we created ‘total’ survival probabilities per patient, for which we summed the five test predictions for each patient (since we repeated our cross-validations five times). Then, we chose the 5-year total survival probability as a timepoint to stratify since this is an often used proxy in cancer survival in the literature. We stratified our KM curves on whether each patient had an above or below the median (across all patients for the cancer type) predicted survival probability at the timepoint closest to 5 years across all test splits, according to each model. KM curves were computed using survival^46^^,^^47^ and survminer^48^ in R.

#### Simulations

Simulated noise modalities were created in Python using np.random.normal. All noise modalities were of dimensionality 10000. Experiments that also contained the true survival time added the observed survival time as a covariate to the clinical modality. The censoring indicator was not used in any way.

#### Reproducibility

All of our results can be reproduced using the code in the GitHub repository https://github.com/BoevaLab/noise_resistance_multi_omics. The repository also contains links to the splits, datasets as well as all of our results.

All deep learning models were trained on a workstation running 16 cores of Intel i9-9900K CPUs @ 3.60GHz. The inner splits (that is, the hyperparameter optimization) for all deep learning methods were parallelized across 16 threads using scikit-learn^49^ and joblib. Statistical models were trained on an internal HPC system instead to speed up computation times. All statistical models except those with noise simulations and with an initial PCA step were run on a node with 34 cores of Intel Xeon (Cascade Lake) CPUs @ 2.60GHz. Statistical models that were part of the noise simulations or that had an initial PCA step were run on a node with 56 cores of AMD EPYC-Rome CPUs @ 2.25GHz. The outer splits for all statistical methods were parallelized across 11 threads using mlr3 and future.^50^

### Quantification and statistical analysis

#### Performance metrics

We used one concordance metric, measuring the discriminative performance of our models, and the IBS to measure both calibration and discrimination of the predicted survival functions. Specifically, we evaluated all models on the survival function scale, computing Antolini’s concordance (Antolini’s C)^51^ using the implementation in *pycox*.^52^ The IBS was also calculated using *pycox*. Since we found the IBS to be highly sensitive to time points close to the end of the test time (presumably due to very low censoring probabilities at these later time points), we evaluated the IBS at 100 uniformly sampled points between the minimum and the 0.9 quantile of observed survival times in each test split, similar to Vale-Silva and Rohr.^21^

Despite this, some deep learning models still had IBS values larger than one on some splits, which were caused by erratic estimated survival functions which led to numerical issues in the calculation of the IBS. We replaced all IBS values larger than one by the maximum for that of all other splits for the same model and dataset. We note that these replacements had no impact on Antolini’s C and noted the number of replacements in Table S5.

We chose Antolini’s C as opposed to Harrell’s C or Uno’s C^53^ due to two reasons.

• The IBS may only be evaluated on the survival function scale and we felt it would not have been fair to then evaluate the concordance measure on the patient-level scale (i.e., risk-measure scale) since the survival function and risk-measure use different estimators for RSF and *BlockForest* (KM and expected mortality respectively).
• Furthermore, in order to make our results comparable to future work, we believed it more robust to evaluate Antolini’s concordance (Antolini’s C), as this will allow for the comparison to models that cannot make risk measure predictions (such as *CoxTime*,^52^ *DeepHit*,^23^ and Logistic Hazard^22^ as, for example, implemented by Vale-Silva and Rohr^21^) in the future, as long as most software packages do not provide robust (or any) transformations from survival to risk-level predictions.^54^

For iterations resulting in model failures (e.g., if there was an error, or the Elastic net selected a completely sparse model, or numerical issues leading to NaN values), we replaced models with a KM estimator on the training set, similar to previous work.^12^ We note that this was only rarely the case except for methods based on glmnet, for which our numbers of failures were consistent with previous work.^12^ We note the number that each model failed (or was completely sparse, for sparse methods) in Table S6.

#### Validation

For all datasets in our experiments, we performed outer 5-fold cross-validation, five times repeated, stratified on the event indicator, giving us a total of 25 test splits per dataset. Hyperparameter tuning details are detailed in the respective sections on statistical and deep learning multimodal integration methods. Splits were performed using the StratifiedKFold implementation of scikit-learn.

For statistical significance testing, we tested for an overall difference between models by adopting paired Wilcoxon signed-ranks test as recommended for comparisons across datasets.^55^^,^^56^ In brief, we calculated the mean across all 25 test splits for each model and cancer type and used the 17 resulting means as inputs to the paired Wilcoxon signed-ranks test. We note that this approach is consistent with previous work.^13^ All tests were performed one-sided in the appropriate direction, with the exception of the comparison to the best unimodal model, which was performed two-sided. Tests comparing each model to the best model per modality choice and metric and comparing each model to the best unimodal model per metric were corrected for multiple testing using the Bonferroni-Holm method.^57^ All other tests were not corrected, since each hypothesis space contained only relatively few tests (since we only compared one model to the same model in a different setting, as opposed to all models to each other). Paired Wilcoxon signed-rank tests were implemented using the wilcox.test function in R and multiple testing correction was performed using the p.adjust function in R.

We emphasize that we deliberately did not test for per-dataset differences between models, as we were interested in an overall assessment of the models (that is, across datasets, as individual datasets may not be representative).

## Data Availability

• All the data reported in this paper have been deposited at Zenodo and are publicly available via the DOI in the key resources table. All raw data used within this paper are accessible via PANCANTALAS and Xenabrowser. We also provide scripts to automatically download and preprocess the raw data via a bash script that is accessible on our Github repository. Links and DOIs are listed in the key resources table. • All original code has been deposited on Github and is publicly available. The Github repository link and archival DOI are listed in the key resources table. • Any additional information required to reanalyze the data reported in this paper is available from the lead contact upon request.

## Supplemental information

Document S1. Figures S1, S2 and Tables S1–S6

Document S2. Article plus supplemental information
