# Supplementary material for: Systematic comparison of multi-omics survival models reveals a widespread lack of noise resistance
Source: Cell Rep Methods. 2023 Apr 24;3(4):100461. doi: 10.1016/j.crmeth.2023.100461 (PMC10162996; doi:10.1016/j.crmeth.2023.100461)
Supplement: Document S1. Figures S1, S2 and Tables S1–S6 [file mmc1.pdf]

Cell Reports Methods, Volume *3*

Supplemental information

Systematic comparison of multi-omics survival models reveals a widespread lack of noise resistance

David Wissel, Daniel Rowson, and Valentina Boeva

Table S1: **Overview of all datasets considered in our work. GEX: gene expression; CNV: copy number variation; RPPA: Reverse Phase Protein Array (RPPA) profiling protein expression. Modality columns denote the dimensionality of that modality in a specific dataset. *N* denotes the number of samples. For full cancer-type abbreviations, please refer to Table S3. Dimensionality is shown per dataset after removing zero variance features. Related to STAR methods.**

Project	Cancer	Clinical	GEX	Mutation	Methylation	CNV	RPPA	miRNA	Modalities	Dimensionality	*N*	#Events
TCGA	BLCA	5	20227	16359	18829	24776	189	740	7	81119	330	149
TCGA	BRCA	5	20227	15380	18829	24776	190	737	7	80144	771	103
TCGA	COAD	5	17507	17576	18821	24776	191	740	7	79616	289	64
TCGA	ESCA	6	19076	9150	18790	24776	193	737	7	72728	123	44
TCGA	HNSC	5	20154	11771	18827	24776	191	733	7	76457	202	122
TCGA	KIRC	4	20223	9329	18808	24776	191	732	7	74063	312	88
TCGA	KIRP	4	20174	8792	18820	24776	194	738	7	73498	203	29
TCGA	LGG	4	20210	10835	18826	24776	190	740	7	75581	412	95
TCGA	LUAD	3	20164	16076	18825	24776	189	738	7	80771	340	136
TCGA	PAAD	5	19955	9477	18784	24776	190	733	7	73920	109	61
TCGA	SARC	4	20209	8437	18782	24776	193	739	7	73140	194	70
TCGA	STAD	4	19076	16853	18817	24776	193	743	7	80462	304	126
TCGA	UCEC	3	17507	19218	18834	24776	191	743	7	81272	395	63
TCGA	OV	3	19064	8483	18770	24776	191	731	7	72018	166	98
TCGA	LIHC	5	20078	8719	18818	24776	190	742	7	73328	157	80
TCGA	LUSC	3	20232	15615	18829	24776	189	739	7	80383	288	117
TCGA	CESC	4	20030	13181	18809	24776	193	737	7	77730	141	24

Table S2: **Mean Antolini's C and IBS across splits and datasets on TCGA on each modality separately using the Elastic net, a deep learning model (*Early*) and an RSF. Standard errors are in parentheses. ↑ denotes a metric for which higher values are better, ↓ the opposite. Related to Table 1.**

	Antolini's concordance							Integrated Brier Score						
	Clinical	GEX	Mutation	Methylation	miRNA	RPPA	CNV	Clinical	GEX	Mutation	Methylation	miRNA	RPPA	CNV
Early	0.627 (0.0059)	0.610 (0.0057)	0.526 (0.0047)	0.592 (0.0058)	0.592 (0.0053)	0.584 (0.0054)	0.550 (0.0052)	0.170 (0.0021)	0.177 (0.0026)	0.195 (0.0028)	0.180 (0.0026)	0.178 (0.0024)	0.177 (0.0023)	0.186 (0.0024)
Elastic net	0.624 (0.0055)	0.586 (0.0058)	0.528 (0.0035)	0.561 (0.0055)	0.573 (0.0057)	0.568 (0.0051)	0.540 (0.0049)	0.168 (0.0021)	0.168 (0.0020)	0.173 (0.0019)	0.171 (0.0021)	0.170 (0.0021)	0.172 (0.0020)	0.173 (0.0020)
RSF	0.609 (0.0056)	0.601 (0.0055)	0.519 (0.0051)	0.568 (0.0060)	0.596 (0.0053)	0.587 (0.0052)	0.554 (0.0049)	0.168 (0.0021)	0.167 (0.0020)	0.181 (0.0020)	0.171 (0.0021)	0.167 (0.0019)	0.169 (0.0019)	0.175 (0.0021)

Table S3: Tissue sites and full names of all cancer abbreviations used throughout our work. Related to STAR methods.

Project	Abbreviation	Tissue	Full name
TCGA	BLCA	Bladder	Bladder Urothelial Carcinoma
TCGA	BRCA	Breast	Breast invasive carcinoma
TCGA	COAD	Colon	Colon adenocarcinoma
TCGA	ESCA	Esophagus	Esophageal carcinoma
TCGA	HNSC	Head and Neck	Head and Neck squamous cell carcinoma
TCGA	KIRC	Kidney	Kidney renal clear cell carcinoma
TCGA	KIRP	Kidney	Kidney renal papillary cell carcinoma
TCGA	LGG	Brain	Brain Lower Grade Glioma
TCGA	LUAD	Lung	Lung adenocarcinoma
TCGA	PAAD	Pancreas	Pancreatic adenocarcinoma
TCGA	SARC	Bones and soft tissue	Sarcoma
TCGA	STAD	Stomach	Stomach adenocarcinoma
TCGA	UCEC	Uterus	Uterine Corpus Endometrial Carcinoma
TCGA	OV	Ovaries	Ovarian serous cystadenocarcinoma
TCGA	LIHC	Liver	Liver hepatocellular carcinoma
TCGA	LUSC	Lung	Lung squamous cell carcinoma
TCGA	CESC	Cervix	Cervical squamous cell carcinoma and endocervical adenocarcinoma

Table S4: **Mean Antolini's C and IBS across splits and datasets on TCGA when integrating clinical data and gene expression compared to integrating all of the available modalities (described in Table S1) with multimodal dropout enabled (see STAR methods for details). Standard errors are in parentheses. † indicates significantly better performance compared to the same setting without multimodal dropout (Table 1). ↑ denotes a metric for which higher values are better, ↓ the opposite. P-values are based on paired one-sided Wilcoxon signed-rank tests (see STAR methods for details). Related to STAR methods.**

	Antolini's concordance ↑		Integrated Brier Score ↓	
	Clinical + GEX	All	Clinical + GEX	All
Early	0.605 (0.0058)	0.584 (0.0060)	0.174 (0.0024)	0.173 (0.0023)
Intermediate (Concat)	0.634 (0.0058)	0.629 (0.0054)†	0.170 (0.0025)	0.180 (0.0028)
Late (Mean)	0.639 (0.0055)†	0.635 (0.0055)†	0.168 (0.0023)	0.171 (0.0024)

Table S5: **Number of times that the Integrated Brier Score (IBS) was imputed using the maximum observed IBS across all splits for the same model, experiment, and dataset. Related to STAR methods. Related to STAR methods.**

Experiment	Modalities	Model	Number of IBS values imputed
Multimodal dropout	All	Intermediate (Concat)	1
Noise (without target)	All	Intermediate (Attention)	3
Noise (without target)	All	Intermediate (Concat)	11
Noise (without target)	All	Intermediate (Mean)	4
Noise (without target)	All	Late (Mean)	8
Noise (without target)	All	Late (MoE)	7
PCA	Clinical + GEX	Intermediate (Concat)	1
PCA	Clinical + GEX	Late (MoE)	1
PCA	All	Early	1
PCA	All	Intermediate (Attention)	1
PCA	All	Intermediate (Max)	2
PCA	All	Intermediate (Mean)	3
PCA	All	Late (Mean)	1
Regular	Clinical + GEX	Intermediate (Attention)	1
Regular	Clinical + GEX	Late (MoE)	1
Regular	All	Intermediate (Concat)	3
Regular	All	Late (MoE)	1

Table S6: **Percentage of runs per model, dataset, and experiment that each model was replaced by a Kaplan-Meier estimator due to a failure of the model or a completely sparse model. Related to STAR methods.**

Experiment	Modalities	Model	Percentage
Multimodal dropout	Clinical + GEX	Intermediate (Concat)	0.5
Multimodal dropout	All	Early	0.9
Multimodal dropout	All	Intermediate (Concat)	0.7
Regular	Clinical + GEX	Intermediate (Concat)	0.5
Regular	Clinical + GEX	Late (MoE)	0.5
Regular	All	Early	0.7
Regular	All	Intermediate (Concat)	1.4
Regular	All	Intermediate (Max)	1.2
Regular	All	Late (MoE)	0.9
Unimodal	Clinical	Early	3.8
Unimodal	CNV	Early	0.2
Unimodal	Methylation	Early	0.2
Unimodal	Mutation	Early	1.2
Regular (with target)	Clinical + GEX	Late (MoE)	0.5
PCA	Clinical + GEX	Late (MoE)	0.1
Noise	All	Early	0.4
Noise	All	Intermediate (Attention)	0.1
Noise	All	Intermediate (Concat)	0.1
Noise	All	Intermediate (Max)	0.0
Noise	All	Intermediate (Mean)	0.0
Noise	All	Late (Mean)	0.2
Noise	All	Late (MoE)	0.6
Regular	Clinical + GEX	Elastic net	28.5
Regular	Clinical + GEX	PriorityLasso	15.3
Regular	All	Elastic net	31.8
Regular	All	PriorityLasso	0.7
Unimodal	Clinical	Elastic net	30.4
Unimodal	CNV	Elastic net	52.2
Unimodal	GEX	Elastic net	29.4
Unimodal	Methylation	Elastic net	40.7
Unimodal	miRNA	Elastic net	28.9
Unimodal	Mutation	Elastic net	84.7
Unimodal	RPPA	Elastic net	30.1
Regular (with target)	Clinical + GEX	PriorityLasso	5.2
PCA	Clinical + GEX	Elastic net	20.9
PCA	Clinical + GEX	PriorityLasso	16.4
PCA	All	Elastic net	25.1
PCA	All	PriorityLasso	1.7
Noise	All	Elastic net	15.5
Noise	All	PriorityLasso	5.2

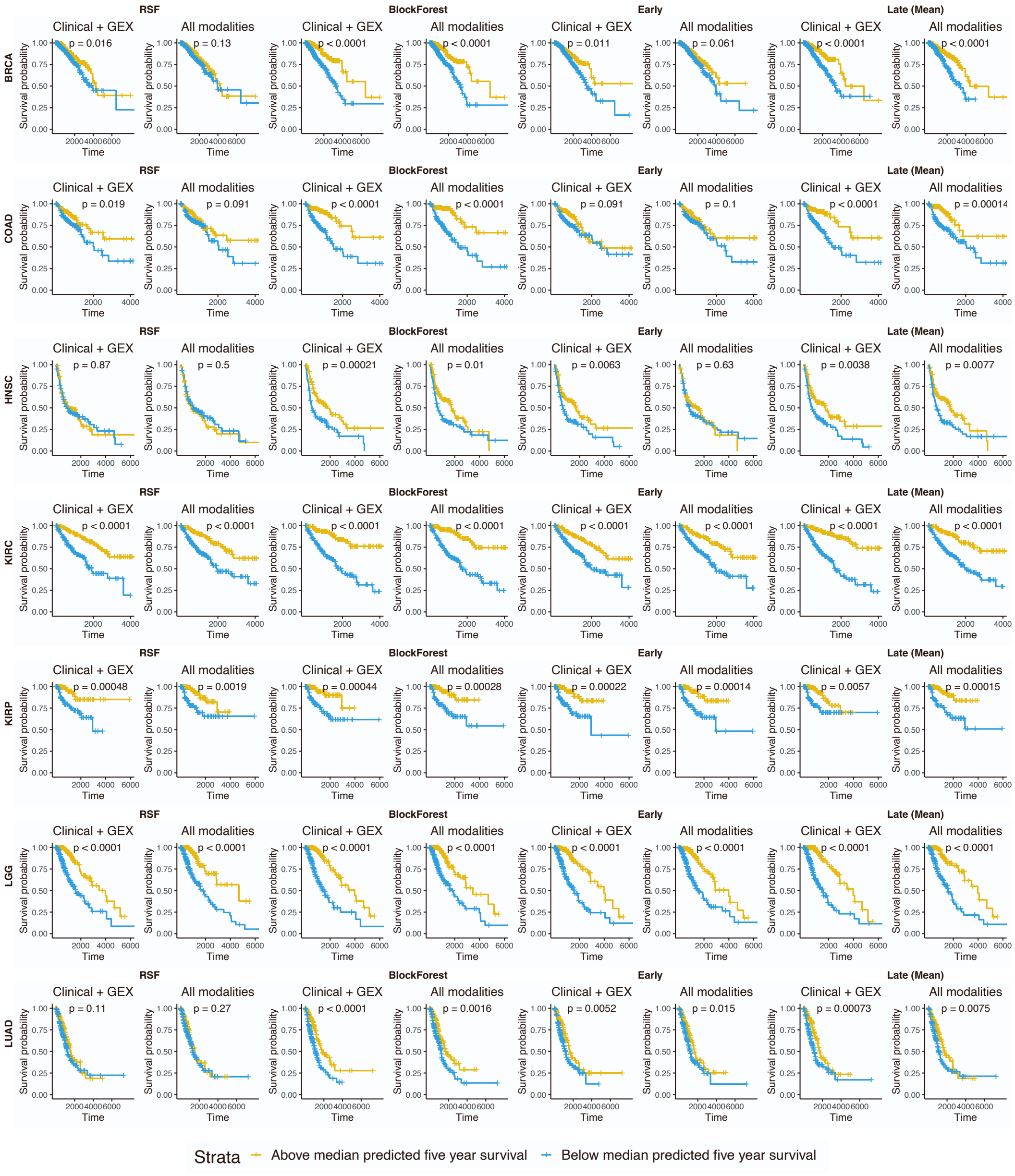

Figure S1: **Kaplan-Meier curves stratified by whether a patient was below or above the median predicted five-year survival according to the model in question (see STAR methods for further details). RSF, *BlockForest*, *Early* and *Late (Mean)* shown on all cancers not contained in Figure 2. P-values are based on log-rank tests. Continued in Figure S2. Related to Figure 2.**

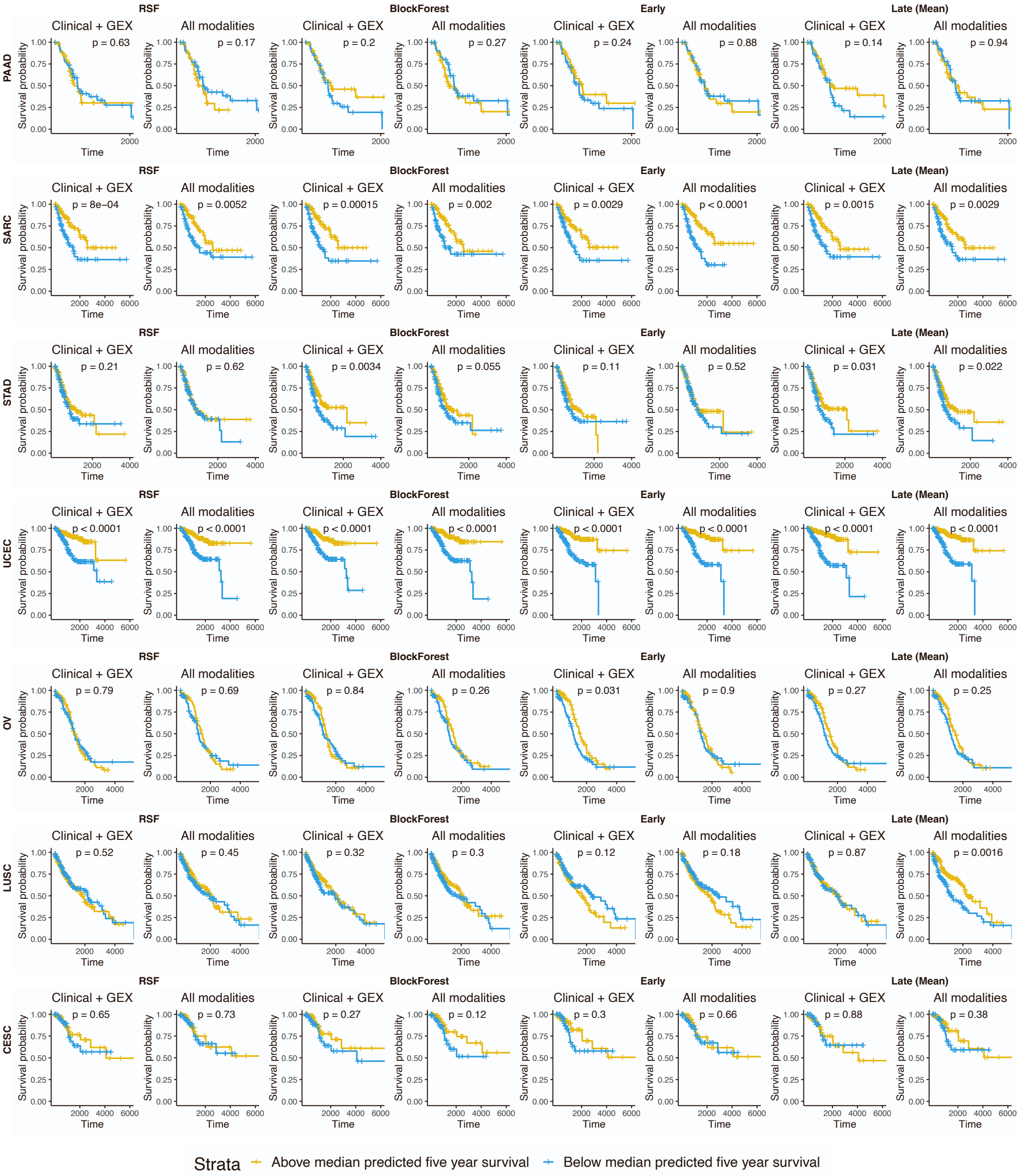

Figure S2: **Kaplan-Meier curves stratified by whether a patient was below or above the median predicted five-year survival according to the model in question (see STAR methods for further details). RSF, *BlockForest*, *Early* and *Late (Mean)* shown on all cancers not contained in Figure 2. P-values are based on log-rank tests. Related to Figure 2.**
